# Factor structure of food and physical activity parenting practices among US fathers by ethnicity and survey language: a cross sectional study

**DOI:** 10.1186/s12889-025-24584-1

**Published:** 2025-10-28

**Authors:** Salma M.A. Musaad, Louise C. Mâsse, Alicia Beltran, Oriana Perez, Teresia M. O’Connor

**Affiliations:** 1 USDA/ARS Children’s Nutrition Research Center, Department of Pediatrics, Baylor College of Medicine, 1100 Bates Street, Houston, TX 77030 USA; 2 School of Population and Public Health, BC Children’s Hospital Research Institute, University of British Columbia, Vancouver, BC Canada

**Keywords:** Parenting practices, Child physical activity, Child feeding, Confirmatory factor analysis

## Abstract

**Background:**

Parenting practices contribute to children’s eating and physical activity behaviors, and thereby their risk of obesity and associated medical conditions. Fathers may engage in different parenting practices than mothers. The objective of this study was therefore to establish psychometrically sound tools in English and Spanish for measuring Physical Activity Parenting Practices (PAPP) and Food Parenting Practices (FPP) for Hispanic and non-Hispanic fathers of diverse races for use in future studies, such as evaluations of family-based obesity interventions targeted at fathers.

**Methods:**

We conducted a preliminary assessment of the psychometric properties of a reduced version of the PAPP and FPP item banks which were administered in English or Spanish to 639 fathers of 5–11 year old children. We used confirmatory factor analyses (CFA) to examine factor structures of the reduced item banks by survey language and ethnicity (Hispanic, non-Hispanic). The internal consistency of the scales were assessed using Cronbach’s alpha.

**Results:**

We identified three factors for PAPP (Autonomy Support, Co-Participation/Modeling, Coercive Control) with two sub-factors each; four factors for FPP (Child Involvement, Covert Control, Modeling, Threats & Bribes) without sub-factors. The solutions performed well in the samples of English and Spanish-speaking fathers. The fit of the FPP factors was adequate among fathers who completed the survey in English, and to a lesser extent among Spanish-speaking fathers, albeit acceptable. A bi-factor model, where each item loads on a primary dimension and supports the use of global factors, was identified for all PAPP scales in both languages with adequate internal consistency and fit. The CFA structure observed by survey language versions was less supported across ethnicities, but did not diverge enough to warrant major modifications. The internal consistency of the FPP Covert Control factor among Hispanics and the Spanish survey sample was low.

**Conclusions:**

This work builds on previously published psychometrics of a large item bank for improving fathers’ measurement of PAPP and FPP. Findings support the use of a reduced version of the parenting practices for fathers when administering the survey in English and Spanish. Further research is needed to investigate the measurement of fathers’ parenting practices across other Spanish speaking groups.

**Supplementary Information:**

The online version contains supplementary material available at 10.1186/s12889-025-24584-1.

## Background

Obesity is a public health concern with multiple risk factors [1], among which inadequate diet and low levels of physical activity (PA) are key [2, 3]. Fathers are important in role-modeling and promoting feeding and PA behaviors for their children [4–8] which can influence children’s risk of developing obesity and associated medical conditions. Yet, observational and interventional research on the role of fathers in this context is understudied, particularly among families traditionally under-represented in research [9–14]. There is a paucity of brief food parenting practices (FPP) or PA parenting practice (PAPP) survey instruments that have been validated for use among fathers of elementary school aged children. Many instruments available are intended for parents of children in younger [15, 16] or older age groups [17]. To inform and expand child obesity research to include more fathers, psychometrically sound instruments are needed to measure food and PA practices among a diverse group of fathers and offered in languages appropriate for the target population. For example, offering surveys in Spanish, in addition to English, engenders inclusiveness for Hispanic fathers. However, differences in underlying constructs from the same survey used in different languages could potentially arise due to measurement difference, self-selection bias, race/ethnicity, and differences in interpretation, culture and demographic characteristics [18, 19]. Improving the measurement of the same constructs among different groups will enhance comparability across surveys irrespective of survey language or ethnicity of the sample, to develop instruments that can be used by a variety of different studies.

We were interested in developing brief psychometrically sound instruments to assess food and PAPPs among fathers participating in family-based obesity-prevention or healthy lifestyle interventions targeted at fathers. Brief surveys to assess FPP and PAPP will help reduce participant burden, which may be particularly important when engaging with fathers in research [20] and to reduce participant burden during randomized controlled trials [21, 22]. In our previous work, we developed conceptual frameworks and items banks of FPP and PAPP using theoretically derived constructs consistent with general parenting practices [23–28]. This previous work assessed the psychometrics of the resulting FPP and PAPPs among a representative sample of 799 and 626 Canadian parents respectively; each included about 50% fathers. The parenting practices mapped across sub-factors in three general parenting domains: Autonomy Promotion, Structure and Control based on expert panel input [25, 26]. Autonomy Promotion was defined as promoting ways for the child to make developmentally appropriate choices and decisions of their own behaviors in the context of being supported by their parent. Structure was a parent’s attempts to organize the child’s environment to promote child proficiency in a non-directive way. Control was the parent’s attempts to dominate or impose their will on the child’s behavior without regard to the child’s desires. The original English language items were assessed by cognitive interviews and demonstrated good psychometric properties, including invariance among the Canadian mothers and fathers [23, 24]. However, while the item banks have shown psychometric stability among diverse parents in Canada, including fathers; the racial and ethnic background of families from the United States is quite different from Canada. For the present study, we focused on the one or two most relevant constructs (factors) within each of the three domains to keep the burden on fathers at a minimum for future evaluations of father-targeted obesity-prevention programs.

The primary objective of this paper was to provide a preliminary assessment of the psychometric properties of a reduced version of the PAPP [24] and the FPP [23] item banks administered to a diverse sample of fathers of 5–11 years old children from the United States. In addition, this study determined whether their factor structure was comparable between the English and Spanish versions, and by self-reported ethnicity (Hispanic, non-Hispanic) (secondary objectives).

## Methods

### Participant recruitment and data collection

The sample for this study was drawn from two different studies: 1) an online study targeting fathers, specifically designed to assess the psychometrics of the food and PA parenting surveys; and 2) the baseline data from Spanish speaking Hispanic fathers who took part in a feasibility study of Papas Saludables Ninos Saludables [29], an intervention targeting Hispanic fathers and children for healthful eating, physical activity and weight. The same FPP and PAPP survey tool was used for both studies.

### Online study (sample 1)

**Fig. 1:**
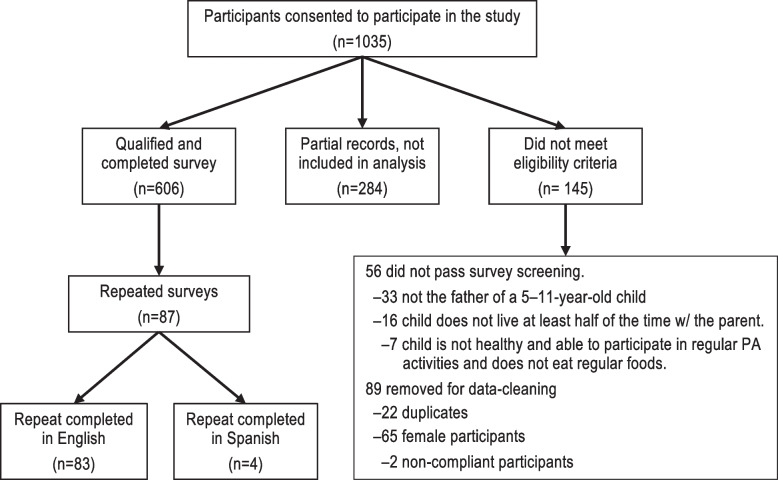
Participant recruitment flowchart

This was a cross-sectional study of fathers of 5–11-year-old children in the United States from April 2018-July 2019. The study aimed to enroll 600 fathers of diverse race and ethnicity to ensure a sample size large enough to conduct confirmatory factor analysis (i.e. 3–10 subjects per item [30] or at least 200 subjects each of Hispanic and non-Hispanic ethnicity for a theoretical Confirmatory Factor Analysis (CFA) model using items on a categorical scale [30]). Fathers were recruited using notices, posters, and flyers posted through local organizations including community centers, health clinics, local fatherhood organizations, local businesses, local TV stations, work sites, child-care centers, schools, the Texas Medical Center, social media, and local community and health related newsletters. Fathers listed on the Children’s Nutrition Research Center volunteer list were contacted by email or a phone call. Interested fathers (*n* = 1949) were directed to the online survey where they could select an English or Spanish version of the study. The study, its risks, data security and how participation was voluntary was explained in a cover letter and fathers were asked if they agreed (consent) to participate. Eligibility was determined based on the following inclusion criteria using an online branching screener: a father of a child between the ages of 5 to 11 years old, the child lived with the father at least 50% of the time, and the child was healthy to participate in regular physical activities (such as school Physical Education classes) and to eat regular foods. All the instructions and questions were written, but also available to be heard as audio in the selected language to assist those with limited literacy. Participants were nominally compensated for their participation ($15 USD). A total of 1035 fathers consented to take part in the study and of these 890 were found to be eligible for this study. Out of the 890 participants, 606 had complete survey data and were included in the analyses, out of which 48 completed the surveys in Spanish language. Fourteen percent (*n* = 87) of the participants who finished the survey completed it a second time within 30 days and were used to assess test–retest reliability. An additional $15 was given to the fathers that completed the survey a second time. Figure 1 presents the recruitment flowchart for the online study (sample 1). This research was approved by the Institutional Review Board at Baylor College of Medicine [protocol H-38237] and meets all requirements for ethical conduct for research with human subjects.

### Papas Saludables Ninos Saludables feasibility study (sample 2)

Hispanic Fathers or father-figures, and up to 3 of their children between the ages of 5–11 years old were recruited from a pediatric clinic via fliers and presentations by study staff in the clinic waiting room. The fathers and their children were recruited from August 2018-December 2018 to participate in a randomized controlled feasibility trial [29] of a culturally adapted version [22] of Healthy Dads Healthy Kids [21, 31]. Of the 36 Hispanic fathers who participated, 33 completed the assessments in Spanish as part of other assessments during the in-person data collection for the feasibility study. Their baseline data were used to enhance the number of surveys completed in Spanish for this analysis. Hence, the total analytic sample used in this study from sample 1 and 2 was 639 of which 81 completed the survey in Spanish. To ensure we obtain reliable estimates in the factor analysis, we oversampled the responses in the surveys that were completed in Spanish. Further details are provided in the Statistical Analysis section which detailed the oversampling of the Spanish language surveys.

### Measures

#### Demographics

Child age, child sex, born in the US, race, marital status, education level, employment, relationship of father to child, and father age were assessed in the same manner in both studies. Annual household income was collected using the following cutoffs: < $25,000, $25,000-$46,999, > $47,000. The lower cutoff was selected based on the poverty threshold of a family of four [32, 33], based on the average Hispanic household of 3.25. The upper cutoff was based on the median income of Hispanic households in 2016 [34] and the threshold for middle class in the United States (US) in 2016 [35].

#### Parenting practices

The parenting practices items were taken from the previously published and psychometrically validated PAPP [24] and FPP [23]. To create a brief version of both the PAPP and FPP to reduce the participant burden for fathers and assess father targeted interventions in RCTs, we elected to focus on the parenting constructs often targeted by child obesity prevention interventions, such as the Healthy Dads Healthy Kids intervention [21] and the culturally adapted Healthy Dads Healthy Kids intervention for Hispanic families [22]. With respect to FPP, we included one construct from the Autonomy Promotion domain that measured Child Involvement (5 items – where 1 item was new); two constructs from the Structure domain that measured Covert Control (4 items), and Modeling (3 items); and one construct within the Control domain that measured Threats and Bribes (7 items – including 1 item (in the past month how often did you take away TV or videogame time if your child did not finish his or her meal??) that was adapted from another existing item (in the past month how often did you threaten to take away TV or videogame time if your child did not eat the vegetables you served?)). The new item came from input from Hispanic fathers who engaged in cognitive interview during formative studies to cultural adapt the Healthy Dads Healthy Kids program for Hispanic fathers and kids [22]. With respect to PAPP, we included items from: 1) the Autonomy Promotion domain that measured Parental Involvement (5 items) and Praise (3 items) which together assessed the Autonomy Support construct; 2) the Structure domain that measured Co-Participation (5 items) and Modeling (5 items) which together assessed the Non-Directive construct; and 3) from the Control domain we included two constructs that measured Guilt (7 items) and Pressure (11 items) which together assessed the Coercive Control construct. One item in the Structure domain belonged to an older version of the Item Bank and was not published previously (item 9: in the past month, how often did you do household chores in front of your child to show him/her you are physically active). Items asked how often the parent performed the practice in the past month. All items were measured on a five-point Likert scale, with responses ranging from “Never” (= 1) to “Always” (= 5).

To construct the Spanish surveys, the items were translated into Spanish and back-translated into English by two separate bilingual staff for content equivalence. Once the surveys were developed in both languages, two rounds of cognitive interviews were conducted with a small sample of Hispanic fathers in English and Spanish. The cognitive interviews resulted in some minor word changes and the addition of one extra FPP item based on cognitive interviews (Item 29: in the past month, how often did you give your child a choice of how to season their fruit or vegetables?).

### Statistical analysis

#### General considerations

Data analysis was conducted on the 639 fathers combined from both samples. Comparisons of categorical data across groups were performed using the Chi square test or Fisher’s exact test. Continuous data were compared across groups using the Wilcoxon two-sample test with *t*-approximation. A two-tailed P value < 0.05 was considered statistically significant. No adjustment for multiple testing was performed in this preliminary investigation. To conduct the factor analysis on the same participants, only participants with complete responses on both parenting practices items were used in this analysis. Analyses were conducted using the Statistical Analysis Software (SAS) version 9.4 (SAS Institute, Cary, NC, USA) or Mplus version 8 [36].

#### Factor structure

CFA was used to assess the factor structure of the parenting practices constructs for the whole group (aim 1, *n* = 639), as well as by the following characteristics (aim 2): survey language- English (*n* = 558) and Spanish (*n* = 243 oversampled from 81 Spanish surveys); self-reported ethnicity- Hispanic (*n* = 261) and non-Hispanic (*n* = 378). This allowed us to validate whether the reduced version administered to the fathers in English and Spanish aligned with the hypothesized PAPP and FPP conceptual framework. CFAs were conducted for each domain of parenting practices and separate CFAs were conducted by language of administration and self-reported ethnicity (Hispanic and non-Hispanic) using the hypothesized structure associated with the PAPP [24] and FPP [23]. Since the PAPP and FPP items were assessed on a 5-point Likert scale, the CFA were conducted using the polychoric correlations estimated with the CORR procedure in SAS version 9.4 (SAS Institute, Inc., Cary, NC). The CFA utilized the maximum likelihood method of estimation, and model fit [37, 38] was determined using the Standardized Root Mean Square Residual (SRMR) (appropriate fit suggested for values of ≤ 0.08), Root Mean Square Error of Approximation (RMSEA) (appropriate fit suggested for values of ≤ 0.08), and the Comparative Fit Index (CFI) (appropriate fit suggested for values of ≥ 0.95). Factors that had correlation coefficient ≥ 0.7 were further evaluated with a confirmatory bi-factor item analysis to examine whether a general latent trait explained the observed correlations among the items, thus supporting collapsing the items into a simpler structure. A bi-factor model, occurs when each item loads on a primary dimension and supports the use of sub-factors [39]. Model fit of the bi-factor item analyses were first evaluated with the CFA model fit indices as well by evaluating the Item Expected Cross-Validation index (I-ECV). I-ECV was calculated as [square of the item loading in the general factor of the bi-factor structure/(square of the item loading in the general factor of the bi-factor structure + square of the item loading in the sub-factor of the bi-factor structure)], where a value greater than 0.50 indicates that the item loads higher onto a general construct and lower values can be maintained if supported conceptually but doing so would highlight a weakness in the bi-factor structure [40]. Modification indices were evaluated when model revisions were indicated. In addition to model fit, models were assessed based on theoretical grounding, cultural application and practical use for future scoring. Standardized Cronbach’s alphas were calculated to assess internal consistency of the factors.

#### Oversampling the Spanish language surveys

One of the secondary aims was to assess the factor structure of the reduced instrument of FPP and PAPP for fathers who completed the Spanish language surveys (*n* = 81) separately from those who completed the English language surveys (*n* = 558). However, the small sample size [41, 42] of fathers who completed the surveys in Spanish language hampered the accuracy of the CFA findings. Thus we decided to use a balanced bootstrap oversampling approach [43] because it resulted in equal representation of the original observations and unbiased estimates to strengthen the confidence in the CFA findings from Spanish respondents. The balanced bootstrap created 3 replicates per participant and increased the sample size from 81 to 243 observations. During this process, the sample observations are selected with replacement and the overall total number of selections is equal across observations. A sample size of 243 was considered sufficient to conduct CFA according to general guidelines (i.e. 3–10 subjects per item) and ratio of items to factors for 4–6 factor solutions [30] or at least 200 subjects for a theoretical CFA model using items on a categorical scale [30]. We assessed the adequacy of the oversampling method by subtracting the oversampled correlation matrix from the original correlation matrix to calculate the standard deviation of their residuals for each item then take the grand mean across all items, a value ≤ 0.1 was considered optimal. The mean was 0 for the oversampled PAPP and FPP Spanish surveys, suggesting that the oversampling method successfully replicated the correlation matrix of the Spanish surveys. Factor scores were calculated as the average of the items in that factor. The scores’ skewness, kurtosis and probability plots were examined to determine the statistical test for comparing between groups. Based on this information, differences in scores between groups were tested using the t-test; significance was determined at the 5% level.

#### Test–retest reliability

Test–retest reliability of factor scores among the 87 participants who took the survey twice within 30 days were computed using the Intraclass Correlation Coefficient (ICC).

## Results

### Description of the sample

Table 1 presents the general characteristics of the study sample by survey language. Out of the 639 responses collected, 558 (87.3%) fathers completed the surveys in English and 81 (12.7%) completed the surveys in Spanish. Compared to participants who completed the surveys in Spanish, those who completed the surveys in English had younger children, fewer were married or living in common law, and had a higher proportion of White, Black/African American and other race, educational attainment and income.

**Table 1:** Characteristics of study sample (*n* = 639)

**Demographics**	**Survey Language**					
	**English**		**Spanish/Espanol**		**Total**	
	**N**	**(%)**	**N**	**(%)**	**N**	**(%)**
All	558	100.00	81	100.00	639	100.00
	**N**	**Mean (SD)**	**N**	**Mean (SD)**	**N**	**Mean (SD)**
Father’s age^**^	558	38.36 (7.99)	81	35.80 (7.20)	639	38.03 (7.94)
Child age^**^						
5–8 years	367	65.77	41	50.62	408	63.85
9–11 year	191	34.23	40	49.38	231	36.15
Child sex						
Boy	307	55.02	49	60.49	356	55.71
Girl	251	44.98	32	39.51	283	44.29
Relationship to child						
Biological father	502	89.96	73	90.12	575	89.98
Stepfather	37	6.63	8	9.88	45	7.04
Adoptive/foster father	14	2.51	0	0.00	14	2.19
Other male relative	5	0.90	0	0.00	5	0.78
Hispanic/Latino^***^						
No	378	67.74	0	0.00	378	59.15
Yes	180	32.26	81	100.00	261	40.85
Race^***^						
White	320	57.35	35	43.21	355	55.56
Black/African American	107	19.18	0	0.00	107	16.74
Mixed/multiple	46	8.24	9	11.11	55	8.61
Asian	38	6.81	1	1.23	39	6.10
Other	37	6.63	35	43.21	72	11.27
American Indian/Alaskan Native	7	1.25	1	1.23	8	1.25
Native Hawaiian/Pacific Islander	3	0.54	0	0.00	3	0.47
Marital status^**^						
Married or common law	479	85.84	79	97.53	558	87.32
Other	79	14.16	2	2.47	81	12.68
Education level^***^						
College graduate/post graduate	303	54.30	5	6.17	308	48.20
Less than HS	6	1.08	30	37.04	36	5.63
Some HS/HS graduate	112	20.07	35	43.21	147	23.00
Technical/some college	137	24.55	11	13.58	148	23.16
Annual household income^***^						
Less than $25,000	65	11.65	31	38.27	96	15.02
$25,000—$46,999	119	21.33	42	51.85	161	25.20
Over $47,000	374	67.03	8	9.88	382	59.78
Primary language spoken in home^*^^*^^*^						
English	449	80.47	1	1.23	450	70.42
Spanish/Espanol	10	1.79	54	66.67	64	10.02
Both English and Spanish	79	14.16	26	32.1	105	16.43
Other	20	3.58	0	0.00	20	3.13

Differences by survey language

*SD* Standard deviation

^**^P < 0.01

^***^P < 0.0001

### Psychometric properties of the FPP

#### Whole sample of fathers (primary objective)

Table 2 depicts the CFA results for the FPP for the whole sample of fathers (*N* = 639). A CFA was conducted for each domain of FPP. Overall, the results replicated the item bank solution with the exception of small modifications made to the Control domain (three items were dropped from the solution). As shown in Table 2, the Autonomy Promotion domain had a one-factor structure which measured Child Involvement (RMSEA = 0.07, CFI = 0.99, SRMR = 0.02), the Structure domain had a two-factor structure measuring Covert Control and Modeling (RMSEA = 0.08, CFI = 0.93, SRMR = 0.05), and the Control domain had a one-factor structure measuring Threats and Bribes (RMSEA = 0.09, CFI = 0.95, SRMR = 0.04). Across all domains, the standardized factor loadings ranged from 0.30–0.84. Noteworthy differences for the FPP results in relation to the hypothesized solution are that: 1) the new item “In the past month how often did you give your child a choice of how to season their fruit or vegetables” was added to the Child Involvement factor in the Autonomy Promotion domain based on cognitive interview findings; 2) three items from the Threats and Bribes factor in the Control domain were dropped as they were correlated with other items and found to be redundant; and 3) the item “In the past month how often did you keep sweets and salty treats out of your child’s reach” on the Covert Control factor in the Structure domain had the lowest loading of 0.30, however it was retained to preserve the content and meaning of the construct. As shown in Table 2, the internal consistency of items, assessed using the Cronbach’s alpha, ranged from 0.60 for the Modeling factor in the Structure domain, to 0.80 for the Threats and Bribes factor in the Control domain. The internal consistency was less than optimal (< 0.70) for three of the four factors.

**Table 2:** Confirmatory factor analysis of the food parenting practices in the whole sample (*n* = 639)

			Whole sample
Autonomy promotion domain In the PAST MONTH,		Factors Cronbach’s α	CFA^1^ ʎ Factors
1	on average how many times did your child help you prepare dinner meals?	Child Involvement (.63)	0.47
2	on average how many times did your child help you prepare vegetable dishes?		0.64
3	on average how many times did you give your child a choice of vegetables to eat at dinner?		0.44
7	how often did you ask your child’s opinion about what to make for meals?		0.41
29*	how often did you give your child a choice of how to season their fruit or vegetables?		0.45
^1^(RMSEA = 0.07 90%CI (0.04–0.11), CFI = 0.99, SRMR = 0.02). *Item 29 was added in this sample based on cognitive interviews with Hispanic fathers			
Structure domain In the PAST MONTH,			CFA^2^ ʎ Factors
9	how often did you keep sweet and salty treats out of your child’s reach?	Covert Control (.62)	0.30
10	how often did you hide soda and sugary drinks in places where your child could not find them?		0.59
14	how often did you throw away left over sweet or salty treats to discourage your child from eating them?		0.61
15	how often did you not bring soda or sweet drinks into your home?		0.66
16	how often did you eat or drink a healthy snack just because your child was around?	Modeling (.60)	0.63
21	how often did you eat healthy portions while in front of your child (for example take a smaller portion)		0.66
23	how often did you show how much you enjoyed eating vegetables while eating with your child?		0.57
	Correlation between Covert Control & Modeling		0.56
^2^(RMSEA = 0.08 90%CI (0.07–0.10), CFI = 0.93, SRMR = 0.05)			
Control domain In the PAST MONTH,		Factors Cronbach’s α	CFA^3^ ʎ Factors
12	how often did you tell your child they will be punished if he or she eats a sweet or salty treat without asking you?	Threats & bribes (.80)	0.58
18	how often did you take away dessert as punishment for bad behavior?		0.66
20	how often did you threaten to send to room if your child refused to eat the vegetables you served?		0.84
24	how often did you tell your child he or she will get dessert only if he or she tasted the vegetables you served?		0.50
26**	how often did you take away TV or videogame time if your child did not finish his or her meal?		0.76
27	how often did you send your child to his or her room if they did not finish their meal?		0.84
28	how often did you reward your child with a sweet or salty treat for good behaviors?		0.49
11	how often did you threaten to take away TV or videogame time if your child did not eat the vegetables you served?	Dropped	High correlation with item 26
19	how often did you offer your child a sweet or salty treat to make your child do something he or she did not want to do?		High correlation with item 28
25	how often did you promise your child dessert if your child finished his or her meal?		High correlation with item 24
^3^RMSEA = 0.09 90%CI (0.08–0.12), CFI = 0.95, SRMR = 0.04. **Item 26 was adapted from item 11			

Item wording has been shortened to conserve space

^†^α = Cronbach’s alpha, ʎ Factor = Standardized factor loadings

#### Comparison of FPP factor structure between English and Spanish versions (secondary objective)

Table 3 shows the FPP CFA results of fathers who completed the survey in English (*n* = 558) and those who completed it in Spanish (*n* = 243 oversampled from 81 fathers). Across both survey languages, the FPP CFA analyses demonstrated adequate support for the hypothesized structure. As shown in Table 3, in the FPP CFA for the English survey language the fit was adequate for the Autonomy Promotion domain (RMSEA = 0.05 90% CI (0.02–0.10), CFI = 0.99, SRMR = 0.02), Structure domain (RMSEA = 0.08 90% CI (0.06–0.11), CFI = 0.93, SRMR = 0.05) and the Control domain (RMSEA = 0.11 90% CI (0.09–0.13), CFI = 0.94, SRMR = 0.05).. As shown in Table 3, in the CFA for the Spanish survey language the fit was adequate for the Autonomy Promotion domain (RMSEA = 0.11 90% CI (0.06–0.17), CFI = 0.97, SRMR = 0.04), Structure domain (RMSEA = 0.12 90%CI (0.09–0.15), CFI = 0.91, SRMR = 0.07) and the Control domain (RMSEA = 0.14 90%CI (0.11–0.17), CFI = 0.92, SRMR = 0.05). While the RMSEA exceeded the optimal value of 0.08 in many cases, the SRMRs were consistently within the optimal range lending support to the retained solution. Evaluation of the item loadings (which ranged from 0.23- 0.93) uncovered that two items from the Covert Control factor in the Structure domain that had sub-optimal loadings – Item 9 “In the past month, how often did you keep sweet and salty treats out of your child’s reach?” factor loading of 0.23 in the English survey sample and item 15 “In the past month, how often did you not bring soda or sweet drinks into your home?” factor loading of 0.22 in the Spanish survey sample. As these two items had high loadings in the other language survey sample, these items were retained to make the factor comparable across survey versions. Internal consistency (Cronbach’s alpha) ranged from low (α = 0.52) to high (0.81), with four out of eight reliability values greater than 0.70.

**Table 3:** Confirmatory Factor Analysis of the food parenting practices stratified by survey language

			**English survey sample (*n* = 558)**	**Spanish survey sample (*n* = 243)**^†^
Autonomy promotion domain In the PAST MONTH,		**Factors** (Cronbach’s α_English_/α_Spanish_)	**CFA**^**1**^ ʎ Factors	**CFA**^**2**^ ʎ Factors
1	on average how many times did your child help you prepare dinner meals?	**Child Involvement** (.62/.71)	0.43	0.69
2	on average how many times did your child help you prepare vegetable dishes?		0.61	0.78
3	on average how many times did you give your child a choice of vegetables to eat at dinner?		0.39	0.83
7	how often did you ask your child’s opinion about what to make for meals?		0.39	0.43
29*	how often did you give your child a choice of how to season their fruit or vegetables?		0.51	0.31
^1^ (RMSEA = 0.05 90%CI (0.02–0.10), CFI = 0.99, SRMR = 0.02); ^2^ (RMSEA = 0.11 90%CI (0.06–0.17), CFI = 0.97, SRMR = 0.04). *Item 29 was added in this sample based on cognitive interviews with Latino fathers				
Structure domain In the PAST MONTH,			**CFA**^**3**^ ʎ Factors	**CFA**^**4**^ ʎ Factors
9**	how often did you keep sweet and salty treats out of your child’s reach?	**Covert Control** (.63/.52)	0.23	0.71
10	how often did you hide soda and sugary drinks in places where your child could not find them?		0.58	0.74
14	how often did you throw away left over sweet or salty treats to discourage your child from eating them?		0.64	0.33
15**	how often did you not bring soda or sweet drinks into your home?		0.69	0.22
16	how often did you eat or drink a healthy snack just because your child was around?	**Modeling** (.56/.78)	0.63	0.69
21	how often did you eat healthy portions while in front of your child (for example take a smaller portion)		0.59	0.93
23	how often did you show how much you enjoyed eating vegetables while eating with your child		0.52	0.75
	**Correlation between Covert Control & Modeling**		0.61	0.46
^3^(RMSEA = 0.08 90%CI (0.06–0.11), CFI = 0.93, SRMR = 0.05); ^4^(RMSEA = 0.12 90%CI(0.09–0.15), CFI = 0.91, SRMR = 0.07) **Items 9 and 15 were retained in the covert control factor of the Structure domain, despite low loading in the English and Spanish survey samples, respectively, to preserve factor content and meaning, and make factor scores comparable across survey versions				
Control domain In the PAST MONTH,		**Factors** (Cronbach’s α_English_/α_Spanish_)	**CFA**^**5**^ ʎ Factors	**CFA**^**6**^ ʎ Factors
12	how often did you tell your child they will be punished if he or she eats a sweet or salty treat without asking you?	**Threats & bribes** (.80/.81)	0.59	0.52
18	how often did you take away dessert as punishment for bad behavior?		0.65	0.77
20	how often did you threaten to send to room if your child refused to eat the vegetables you served?		0.83	0.88
24	how often did you tell your child he or she will get dessert only if he or she tasted the vegetables you served?		0.49	0.58
26***	how often did you take away TV or videogame time if your child did not finish his or her meal?		0.76	0.79
27	how often did you send your child to his or her room if they did not finish their meal?		0.84	0.82
28	how often did you reward your child with a sweet or salty treat for good behaviors?		0.50	0.39
11	how often did you threaten to take away TV or videogame time if your child did not eat the vegetables you served?	**Dropped**	High correlation with item 26	
19	how often did you offer your child a sweet or salty treat to make your child do something he or she did not want to do?		High correlation with item 28	
25	how often did you promise your child dessert if your child finished his or her meal?		High correlation with item 24	
^5^ (RMSEA = 0.11 90%CI(0.09–0.13), CFI = 0.94, SRMR = 0.05); ^6^ (RMSEA = 0.14 90%CI(0.11–0.17), CFI = 0.92, SRMR = 0.05). *** Item 26 was adapted from item 11				

Item wording has been shortened to conserve space

^†^Eighty-one surveys administered in Spanish were oversampled using the balanced bootstrap method at an inflation proportion of 3x (i.e. 3 replicates per participant ID), leading to a final sample size of 243. α = Cronbach’s alpha, ʎ Factor = Standardized factor loadings

#### Comparison of FPP factor structure by self-reported ethnicity (Hispanic, non-Hispanic) (secondary objective)

Additional File 1 presents the CFA of the FPP domains across ethnicities. The CFA was conducted across all domains that were tested in the whole sample. Items that were removed from the whole sample are not included in this table. As shown in Additional File 1, survey responses among those reported to be Hispanic and non-Hispanic had similar model fit, that was deemed to be acceptable as observed by the SRMR which were constantly within acceptable range and the CFI which were within or near acceptable range. For example, in the Hispanic sample, the RMSEA ranged from 0.08 (Control domain) to 0.14 (Autonomy Promotion domain); CFI ranged from 0.91 (Structure domain) to 0.96 (Control domain); the SRMR ranged from 0.04 (Autonomy Promotion and Control domains) to 0.06 (Structure domain). In addition, all factor loadings for these solutions ranged from 0.30- 0.79 among Hispanics, and 0.34 to 0.88 among non-Hispanics indicative of moderate to strong correlation among the factors and the items [44]. Internal consistencies ranged from low (α = 0.52) to high (α = 0.82), with six out of eight values observed to be < 0.70. The internal consistency of the Covert Control factor of the Structure domain was low (0.52) among Hispanics (Additional File 1) as well as the Spanish survey sample (Table 3), suggesting that more items are needed to strengthen this construct among Spanish speakers who identify as Hispanic.

### Psychometric properties of the PAPP

#### Whole sample of fathers (primary objective)

Table 4 shows the CFA results for each domain of the PAPP in the whole sample, indicating that the fit of the models were reasonable. The RMSEA ranged from 0.11 (Control domain) to −0.13 (Autonomy promotion); the CFI ranged from 0.90 (Structure domain) to 0.96 (Autonomy promotion); and the SRMR ranged from 0.04 (Autonomy promotion domain) to 0.06 (Structure domain). However, the CFA solutions for each domain of PAPP were more complex than hypothesized. As shown in Table 4, all CFAs resulted in two sub-factors when only one-factor was hypothesized for all domains. In all cases the two sub-factors from these solutions were highly correlated, with values that ranged from 0.83–0.88, which suggested that a global factor was likely possible, so bi-factor analyses were tested for each domain. The bi-factor solutions showed improved and adequate fit indicating that the hypothesized solutions were replicated with the inclusion of minor dimensions (RMSEA 0.04–0.09, CFI 0.96–0.99, SRMR 0.01–0.03). The I-ECV values ranged from 0.34–1.00 and four items had I-ECV < 0.50, one in the Autonomy Promotion domain (in the past month, how often did you teach your child sport or physical activity skills?); two in the Structure domain (in the past month, how often did you play ball or sports with your child?; select the best answer for you: I tell my child how much I like to exercise or be physically active); one in the Control domain (Select the best answer for you: When the weather allows, I force my child to play outside even if he or she does not feel like it). This finding could be related to loading higher on the sub-factors than on the global factor. All items were kept in the final solution to ensure content representation within the respective domain. Internal consistency of the global factors were all high and ranged from 0.85- 0.90.

**Table 4:** Confirmatory factor analysis of the physical activity parenting practices in the whole sample (*n* = 639)

		**Factors** Cronbach’s α	**Sub-Factors** Cronbach’s α	**Whole sample**	
Autonomy promotion domain In the PAST MONTH,				**CFA**^**1**^ ʎ Factors	**Bi-Factor**^**2**^ I-ECV*
3	how often did you make your child’s sport or physical activity a conversation topic?	**Autonomy Support**.85	**Involvement**.75	0.77	0.80
5	how often did you teach your child sport or physical activity skills?			0.82	0.45
8	how often did you watch your child practice a sport or physical activity?			0.79	0.84
36	I find it stimulating to hear my child talk about the progress he/she is making in learning a new sport or physical activity skill			0.51	0.98
10	how often did you praise your child for being physically active or for taking part in sports or physical activity classes?		**Praise**.82	0.85	0.93
11	how often did you tell your child he or she is doing well in his or her physical activity or sports?			0.91	0.96
4	how often did you help with your child’s organized sports or physical activity as a volunteer?	**Dropped**	**Dropped**	Low loading	
6	how often did you ask your child about his or her physical activity or sport participation?			Item 6 was in the Involvement sub-factor but cross-loaded on Praise	
	**Correlation between Involvement & Praise**			0.88	
^1^(RMSEA = 0.13 90%CI (0.11–0.16), CFI = 0.96, SRMR = 0.04); ^2^ (RMSEA = 0.04 90%CI (0.00–0.08), CFI = 0.99, SRMR = 0.01)					
Structure domain In the PAST MONTH,				**CFA**^**3**^ ʎ Factors	**Bi-Factor**^**4**^ I-ECV*
1	how often did you play ball or sports with your child?	**Co-Participation/Modeling**.88	**Co-Participation**.82	0.80	0.48
2	how often did you ask your child to be active with you?			0.88	0.64
7	how often did you walk or bike with your child to go to places that are near your home even though it would be quicker to drive?			0.62	0.91
12	how often did you arrange for your child to be with friends that would encourage your child to be physically active?			0.61	0.96
17	Select the best answer for you: Our family is physically active together			0.76	0.94
9	how often did you do household chores in front of your child to show him/her you are physically active? (New)		**Modeling**.79	0.48	1.00
13	how often did you do at least 30 min of physical activity or exercise (e.g., walking, cycling, or playing a sport) on your own or with others?			0.70	1.00
20	Select the best answer for you: I am physically active in front of my child			0.84	1.00
29	Select the best answer for you: I talk about my physical activity with my child			0.76	0.85
33	Select the best answer for you: I tell my child how much I like to exercise or be physically active			0.74	0.34
	**Correlation between Co-participation & Modeling**			0.83	
^3^(RMSEA = 0.12 90%CI (0.11–0.14), CFI = 0.90, SRMR = 0.06); ^4^(RMSEA = 0.09 90%CI (0.08–0.10), CFI = 0.97, SRMR = 0.03)					
Control domain Select the best answer for you:				**CFA**^**5**^ ʎ Factors	**Bi-Factor**^6^ I-ECV*
16	I try to guilt my child to be more physically active by telling him/her that he or she has been lazy	**Coercive Control **.90	**Guilt**.81	0.86	0.99
21	I tell my child he or she will gain weight if he or she doesn’t exercise			0.70	0.70
32	I show my child people who are overweight as a way to get him or her to be more physically active			0.74	0.54
34	To make my child do more physical activity, I tell him or her to stop being lazy			0.88	0.88
15	I threaten to take away privileges (e.g., TV or video game times) if my child is not physically active in his or her free time		**Pressure**.87	0.72	0.91
18	I promise my child a sweet or salty treat (e.g., dessert) if he or she is active			0.59	1.00
19	I complain to my child when he or she is not active enough			0.76	0.96
24	The only way I can get my child to play outside is by insisting that my child goes outside			0.75	0.76
25	I get upset and angry at my child if he or she is not physically active in his or her free time			0.86	0.84
26	I have to push my child hard to get better at sports or physical activity skills			0.68	0.66
27	When the weather allows, I force my child to play outside even if he or she does not feel like it			0.72	0.47
35	The only way I get my child to be physically active in his or her free time is by forcing him/her to be active			0.86	0.87
14	I have to constantly remind my child to be physically active in his or her free time	**Dropped**	**Dropped**	Low loading	
22	I tell my child he or she will get diabetes or other diseases if he or she is not physically active			Correlated with item 21	
23	I take something away (like no dessert or TV) or add a chore (like pick up toys) if my child refuses to take part in physical activity or sports			Low loading	
28	I discipline my child for refusing to exercise or being inactive in his or her free time			Low loading	
30	To get my child to practice his or her activities, I often say “your friends will make fun of you if you do not get better”			Low loading	
31	I insist my child participates in organized sports or physical activities instead of playing with his or her friends			Low loading	
	**Correlation between Guilt & Pressure**			0.86	
^5^(RMSEA = 0.11 90%CI (0.09–0.12), CFI = 0.92, SRMR = 0.05); ^6^(RMSEA = 0.09 90%CI (0.08–0.10), CFI = 0.96, SRMR = 0.03)					

α = Cronbach’s alpha; ʎ Factor = Standardized factor loadings; I-ECV = Explained Common Variance for a given Item, where I-ECV greater than. 50 indicated the item loaded on the general domain

Item wording has been shortened to conserve space

*Items with I-ECV < 0.5 were kept because of their importance from a content perspective and contextual fit within the respective domain, removing the item did not impact reliability

#### Comparison of factor structure between English and Spanish versions (secondary objective)

Table 5 presents the CFA results of the PAPP domains among those who completed the surveys in English (*n* = 558) and those who completed the survey in Spanish (*n* = 243 after oversampling from 81 Spanish surveys). The solutions observed with the whole sample were replicated in these sub-samples. Specifically, two-factor solutions had adequate fits for each domain although one-factor solutions were expected; however, in all cases the two-factors for all domains were highly correlated (ranged from 0.82–0.87 in the English survey version and from 0.88–0.94 in Spanish survey) supporting the use of global factors. The bi-factor solutions supported simpler structures that aligned with the hypothesized solution. As shown in Table 5 for the English survey sample, the bi-factor solutions demonstrated excellent model fit, where the RMSEA ranged from 0.04 (Autonomy Promotion domain) to 0.09 (Structure and Control domains); CFI ranged from 0.96 (Structure and Control domains) to 0.99 (Autonomy Promotion domain); and SRMR ranged from 0.01 (Autonomy Promotion domain) to 0.04 (Structure domain). Table 5 also shows the bi-factor solutions for the Spanish survey sample, where the RMSEA ranged from 0.00 (Autonomy Promotion domain) to 0.13 (Structure domain); CFI 0.97 (Control domain) to −1.00 (Autonomy Promotion domain); SRMR 0.00 (Autonomy Promotion domain) to 0.05 (Control domain), reflecting that global factors were supported. The I-ECV were all above 0.50 except for four items, three of which were in the English survey sample and one in the Spanish survey sample. Thus, given that I-ECV values lower than 0.50 for an item were observed in a single sample rather than both, this supported keeping the item in both samples for comparison purposes and to maintain the breadth of the content. Internal consistencies were all high for the global factors and ranged from 0.83–0.89 in the English survey and from 0.89–0.92 in the Spanish survey.

**Table 5:** Confirmatory Factor Analysis of the physical activity parenting practices stratified by survey language

		**Factors** (Cronbach’s α_English_/α_Spanish_)	**Sub-Factors** (Cronbach’s α_English_/α_Spanish_)	**English survey sample (*n* = 558)**		**Spanish survey sample (*n* = 243**^†^	
**Autonomy Promotion domain** In the PAST MONTH,				**CFA**^**1**^ ʎ Factors	**Bi-Factor**^**2**^ I-ECV*	**CFA**^**3**^ ʎ Factors	**Bi-Factor**^**4**^ I-ECV*
3	how often did you make your child’s sport or PA a conversation topic?	**Autonomy Support**.83/.89	**Involvement**.73/.78	0.74	0.77	0.87	0.66
5	how often did you teach your child sport or physical activity skills?			0.81	0.37	0.87	0.79
8	how often did you watch your child practice a sport or physical activity?			0.79	0.81	0.84	0.95
36	Select the best answer for you: I find it stimulating to hear my child talk about the progress he/she is making in learning a new sport or physical activity skill			0.52	0.97	0.46	1.00
10	how often did you praise child for being active or for taking part in sports		**Praise**.78/.94	0.83	0.94	0.94	1.00
11	how often did you tell your child he or she is doing well in his or her physical activity or sports?			0.88	0.94	0.99	1.00
4	how often did you help with your child’s organized sports or physical activity as a volunteer?	**Dropped**	**Dropped**	Low loading			
6	how often did you ask your child about his or her physical activity or sport participation?			Item 6 was in the Involvement sub-factor but cross-loaded on Praise			
	**Correlation between Involvement & Praise**			0.87		0.91	
^1^(RMSEA = 0.14 90%CI (0.11–0.16), CFI = 0.97, SRMR = 0.05); ^2^ (RMSEA = 0.04 90%CI (0.00–0.09), CFI = 0.99, SRMR = 0.01) ^3^ (RMSEA = 0.14 90%CI (0.10–0.18), CFI = 0.99, SRMR = 0.02); ^4^(RMSEA = 0.00 90%CI (0.00–0.08), CFI = 1.00, SRMR = 0.00). *Items with I-ECV < 0.5 were kept because of their importance from a content perspective and contextual fit within the respective domain and removing the item did not impact reliability							
**Structure domain** In the PAST MONTH,				**CFA**^**5**^ ʎ Factors	**Bi-Factor**^**6**^ I-ECV*	**CFA**^**7**^ ʎ Factors	**Bi-Factor**^**8**^ I-ECV*
1	how often did you play ball or sports with your child?	**Co-Participation/Modeling**.87/.89	**Co-Participation**.81/.83	0.79	0.45	0.89	0.73
2	how often did you ask your child to be active with you			0.88	0.64	0.85	0.55
7	how often did you walk or bike with your child to go to places that are near your home even though it would be quicker to drive?			0.62	0.91	0.57	0.73
12	how often did you arrange for your child to be with friends that would encourage your child to be physically active?			0.58	0.95	0.82	1.00
17	Select the best answer for you: Our family is physically active together			0.78	0.91	0.66	1.00
9	how often did you do household chores in front of your child to show him/her you are physically active (New)		**Modeling**.77/.83	0.44	1.00	0.57	0.95
13	Do at least 30 min of physical activity or exercise (e.g., walking, cycling, or playing a sport) on your own or with others?			0.70	1.00	0.71	1.00
20	Select the best answer for you: I am physically active in front of my child			0.85	0.99	0.82	1.00
29	Select the best answer for you: I talk about my physical activity with my child			0.73	0.71	0.84	0.36**
33	Select the best answer for you: I tell my child how much I like to exercise or be physically active			0.71	0.47	0.84	0.94
	**Correlation between Co-participation & Modeling**			0.82		0.88	
^5^(RMSEA =.0.12 90%CI (0.11–0.14), CFI = 0.90, SRMR = 0.06); ^6^(RMSEA = 0.09 90%CI (0.08-.0.11), CFI =.0.96, SRMR = 0.04) ^7^(RMSEA = 0.17 90%CI (0.15–0.19), CFI = 0.94, SRMR = 0.06); ^8^(RMSEA = 0.13 90%CI (0.11–0.15), CFI = 0.98, SRMR = 0.04). *Items with I-ECV < 0.5 were kept because of their importance from a content perspective and contextual fit within the respective domain and removing the item did not impact reliability. **Factor loading for item 29 in the Spanish survey exceeded 1.00 for the bi-factor solution and was set at.99 for the I-ECV computation							
**Control domain** Select the best answer for you:				**CFA**^**9**^ ʎ Factors	**Bi-Factor**^**10**^ I-ECV	**CFA**^**11**^ ʎ Factors	**Bi-Factor**^**12**^ I-ECV
16	I try to guilt my child to be more physically active by telling him/her that he or she has been lazy	**Coercive Control**.89/.92	**Guilt**.81/.81	0.86	0.96	0.86	0.97
21	I tell my child he or she will gain weight if he or she doesn’t exercise			0.71	0.57	0.72	0.98
32	I show my child people who are overweight as a way to get him or her to be more physically active			0.75	0.57	0.66	0.86
34	To make my child do more physical activity, I tell him or her to stop being lazy			0.87	0.81	0.89	0.69
15	I threaten to take away privileges (e.g., TV or video game times) if my child is not physically active in his or her free time		**Pressure**.86/.89	0.73	0.98	0.68	0.99
18	I promise my child a sweet or salty treat (e.g., dessert) if he or she is active			0.58	0.98	0.71	0.99
19	I complain to my child when he or she is not active enough			0.76	0.99	0.79	0.94
24	The only way I can get my child to play outside is by insisting that my child goes outside			0.77	0.88	0.65	0.59
25	I get upset and angry at my child if he or she is not physically active in his or her free time			0.86	0.92	0.91	0.98
26	I have to push my child hard to get better at sports or physical activity skills			0.66	0.72	0.77	0.87
27	When the weather allows, I force my child to play outside even if he or she does not feel like it			0.72	0.53	0.77	0.87
35	The only way I get my child to be physically active in his or her free time is by forcing him/her to be active			0.85	0.93	0.92	0.91
14	I have to constantly remind my child to be physically active in his or her free time	**Dropped**	**Dropped**	Low loading		Low loading	
22	I tell my child he or she will get diabetes or other diseases if he or she is not physically active			Correlated with item 21 and item did not load on the general factor		Correlated with item 21 and item did not load on the general factor	
23	I take something away (like no dessert or TV) or add a chore (like pick up toys) if my child refuses to take part in physical activity or sports			Low loading		Low loading	
28	I discipline my child for refusing to exercise or being inactive in his or her free time			Low loading		Low loading	
30	To get my child to practice his or her activities, I often say “your friends will make fun of you if you do not get better”			Low loading		Low loading	
31	I insist my child participates in organized sports or physical activities instead of playing with his or her friends			Low loading		Low loading	
	**Correlation between Guilt & Pressure**			0.86		0.94	
^9^(RMSEA = 0.11 90%CI (0.09–0.12), CFI = 0.92, SRMR = 0.05); ^10^(RMSEA = 0.09 90%CI (0.08–0.11), CFI = 0.96, SRMR = 0.03); ^11^(RMSEA = 0.11 90%CI (0.09–0.134), CFI = 0.97, SRMR = 0.05); ^12^(RMSEA = 0.12 90%CI (0.11–0.14), CFI = 0.97, SRMR = 0.05							

Item wording has been shortened to conserve space

^†^ Eighty-one surveys administered in Spanish were oversampled using the balanced bootstrap method at an inflation proportion of 3x (i.e. 3 replicates per participant ID), leading to a final sample size of 243. α = Cronbach’s alpha; ʎ Factor = Standardized factor loadings; I-ECV = Explained Common Variance for a given Item, where I-ECV greater than. 50 indicated the item loaded on the general domain and items with lower I-ECV were kept if it made sense conceptually and was supported in one of the samples

#### Comparison of PAPP factor structure by self-reported ethnicity (Hispanic, non-Hispanic) (secondary objective)

Additional File 2 presents the CFA of the PAPP across ethnicities. Similar to the previous CFA analyses, two-factor solutions were found adequate for all domains of PAPP (all SRMRs were within acceptable ranges and variations found for the other indices). As shown in Additional File 2, in all cases, the two-factors were highly correlated (i.e., 0.78 to 0.91), and a global factor (or a bi-factor solution) resulted in improved fit, except for the PAPP Control domains. In the Hispanic sample PAPP Control domain the fit for the two-factors (RMSEA = 0.13, CFI = 0.89, SRMR = 0.05) was better than for the bi-factor solution (RMSEA = 0.17, CFI = 0.87, SRMR = 0.06). A slight deterioration of the fit indices in the PAPP Control domains was also witnessed in the non-Hispanic sample (two-factors (RMSEA = 0.12, CFI = 0.91, SRMR = 0.06) vs. bi-factor (RMSEA = 0.14, CFI = 0.90, SRMR = 0.07)). As the bi-factor for the Hispanic sample’s Structure domain resulted in an improper solution, we tested a one factor solution with correlated error terms between items 1 and 2, items 29 and 33, and items 17 and 20 (Additional File 3). The model produced adequate fit among the Hispanic sample (RMSEA = 0.07, CFI = 0.97, SRMR = 0.03); when tested in the non-Hispanics sample for comparability we also observed adequate fit (RMSEA = 0.11, CFI = 0.93, SRMR = 0.05) (Additional File 3). Due to the observed complex solution, this finding supported the use of the PAPP Structure domain as a one-factor solution in future studies. As shown in Additional File 2, for the two domains where a bi-factor was adequate (Autonomy Promotion and Control domains), the I-ECV values for the bi-factor solutions were low (< 0.5) in the Non-Hispanic sample for one item in the Autonomy Promotion domain (item 5, I-ECV = 0.28) and one item in the Control domain (item 27, I-ECV = 0.49). I-ECV values for the bi-factor solutions were high (> 0.5) for several items, achieving a value of i 1.00 in the Non-Hispanic sample for one item in the Autonomy Promotion domain (item 36) and one in the Control domain (item 18), and in the non-Hispanic sample for one item in the Control domain (item 25). The internal consistency for the global factors ranged from 0.86–0.91 for the Hispanic sample of fathers and ranged from 0.83–0.86 for the non-Hispanic sample of fathers.

### Distribution of parenting practices scores by survey language

Table 6 depicts the distribution of the FPP and PAPP scores among the 558 fathers who completed the survey in English, the original sample of 81 fathers who completed the survey in Spanish, and in the combined sample of 639 fathers. Significant differences between survey languages were noted in the Autonomy Promotion domain (child involvement) for both the FPP and PAPP, with higher scores among fathers who completed the surveys in English compared to fathers who completed the surveys in Spanish. Fathers who completed the surveys in English also had higher scores in the Autonomy Promotion domain (praise) and the Structure domain (Co-participation, modeling) (*P* < 0.0001), compared to those who completed it in Spanish.

**Table 6:** Summary of parenting practices scores overall stratified by survey language and in the whole sample

	**Survey sample**					**Combined sample**	
	**English (*n* = 558)**		**Spanish (*n* = 81)**		**(*n* = 639)**		
	**Mean**	**(SD)**	**Mean**	**(SD)**	**Mean**		**(SD)**
Food parenting practices							
Autonomy Promotion domain							
Child Involvement factor***	2.82	0.68	2.59	0.73	2.79		0.69
Structure domain							
Covert control factor	3.02	0.92	2.87	0.8	3.00		0.90
Modeling factor	3.21	0.81	3.05	1.04	3.19		0.85
Control domain							
Threats & Bribes factor	2.08	0.71	1.96	0.69	2.06		0.70
Physical activity parenting practices							
Autonomy Promotion domain							
Autonomy Support factor							
Child Involvement sub-factor***	3.71	0.83	3.27	0.99	3.66		0.86
Praise sub-factor***	4.13	0.88	3.52	1.29	4.05		0.96
Structure domain Co-participation/Modeling factor							
Co-participation sub-factor***	3.39	0.81	3.00	0.89	3.34		0.83
Modeling sub-factor***	3.59	0.75	3.00	0.95	3.51		0.80
Control domain Coercive Control factor							
Coercive control sub-factor^†^	1.78	0.68	1.81	0.76	1.78		0.69

*SD* Standard deviation

^†^Coercive control is the average of the items that were included in the guilt and pressure factors

^*^P < 0.05, ^**^P < 0.01, ^***^P < 0.0001 for difference between survey language using the t-test

#### Test–retest reliability in sample 1

Table 7 presents the ICC for the FPP and PAPP scores from sample 1 participants who completed the surveys two times, respectively. The ICC among the parenting practices constructs was moderate to high and ranged from 0.53–0.76 for the FPP, and 0.68–0.86 for the PAPP.

**Table 7:** Test–retest reliability of food and physical activity parenting practices scores in sample 1 (*n* = 87)

	**ICC of parenting practices between first and second survey administration**
Food parenting practices scores in the second survey administration	
Autonomy Promotion domain	0.72**
Structure domain-Covert Control factor	0.68**
Structure domain-Modeling factor	0.53**
Control domain-Threats and Bribes factor	0.76**
Physical activity parenting practices scores in the second survey administration	
Autonomy Promotion domain -Involvement sub-factor	0.81**
Autonomy Promotion domain -Praise sub-factor	0.68**
Structure domain -Co-participation sub-factor	0.84**
Structure domain -Modeling sub-factor	0.83**
Control domain -Guilt sub-factor	0.83**
Control domain -Pressure sub-factor	0.86**

## Discussion

This study further expands our ongoing research to develop psychometrically sound self-report tools to measure PAPP and FPP grounded in general parenting conceptual frameworks, this time with an emphasis on fathers. Moreover, this study adds to the limited number of studies that examine the psychometric properties across ethnic groups [45]. Brief and psychometrically sound tools are critical to evaluate child-obesity prevention interventions that engage fathers to better understand if the intervention prompted change in father’s use of parenting practices; and whether father’s FPP and/or PAPP mediate any outcomes for children who participate in such programs. A reduced number of constructs and items from the original item banks of PAPP [24] and FPP [23] were assessed among a diverse sample of fathers from the US to determine the psychometric properties among the new sample. This will help reduce the burden of participating fathers in future studies. The results of the CFAs and bifactor analyses generally support the overall framework for both the reduced PAPP and FPP instruments when administered in English or Spanish. Additionally, the frameworks were also generally supported for Hispanic and non-Hispanic fathers. It is noted that emphasis was placed on not deviating markedly from the conceptual framework grounded in general parenting concepts developed by researchers from our group [25, 26] and others [46]. Slightly lower fit indices or internal consistencies were accepted for some factors to retain the original framework. For example, we observed internal consistencies < 0.70 for the FPP Autonomy Promotion and Structure domains in the whole sample as well as in the English survey sample, but only among the Covert Control factor of the Structure domain in the Spanish survey sample. We also observed a similar finding in the FPP Autonomy Promotion and Structure domains among both ethnicities. Such sub-optimal consistency values could indicate lack of variability among the items and requires further investigation, such as using the longer scales from the original item bank. Although we did not test full invariance of the items in this study, comparisons of the factors’ loadings suggest that full invariance may not be supported. For example, the loadings on the FPP child involvement factor are higher in the Spanish survey sample than the English survey sample for 4 out of 5 items (Table 3), and the loadings for the PAPP Modeling sub-factor are higher in Hispanic sample than the non-Hispanic sample for 4 out of the 5 items (Additional File 2). These observations suggest that the scale scores may not have the same interpretation across groups – meaning that someone with a similar scale score may not have the same response pattern. Our results align with previous validation studies of parenting practices that have examined measurement invariance (equivalence) across groups. For example, weak to no evidence for measurement invariance by ethnicity (Hispanic vs non-Hispanic) was found for the Child Feeding Questionnaire [47], the Parenting Involvement Inventory [48], and the Fathers’ Caregiving and Nurturing scale [49]. Thus, it is still challenging to identify parenting related scales that are fully comparable across Hispanics and non-Hispanics.

Others have assessed the psychometrics of established parenting practices among samples of Hispanic parents [50–52], but often have not been able to support the original factor structure [50, 51]. For example, Arlinghaus et al. [50] assessed the twelve-factor Comprehensive Feeding Practice Questionnaire, originally developed in a primarily non-Hispanic white sample of parents [53], and found that the factor structure was not confirmed in the new Hispanic sample. Instead, a five-factor structure with a reduced number of items emerged in an exploratory factor analysis to have an adequate fit. Anderson et al. [51] assessed the psychometrics of the Child Feeding Questionnaire [54] among Black and Hispanic parents and demonstrated the factor structure was confirmed but revealed conceptual problems with some of the items on the restriction and perceived child weight factors that required modifications to the original factor model. Similarly, Wood et al. [52] assessed the psychometrics of the Infant Feeding Styles Questionnaire [55] among Hispanic parents and found that only three (pressuring, restrictive and responsive) of the original five constructs (laissez faire, pressuring, restrictive, responsive and indulgence) performed well in the CFA. It is noteworthy that in all three of these examples, the instrument was provided in English and Spanish, but except for some sensitivity analysis in the Wood et al. paper [52], the issue of language of the instrument was not addressed. This is concerning because there may be differences in underlying constructs measured due to minor differences in translation, differences in interpretation of items, or culture [18, 19]. It is likely that sample size differences in the fathers who completed the surveys in English and Spanish created barriers in assessing the factor structure by language in addition to ethnicity. In this study we overcame this problem by using a balanced bootstrap oversampling method [43] which allowed us to explore the factor structure for both different languages and self-reported ethnicity.

A key strength of the original (previous) psychometric evaluations of the FPP and PAPP is that the sample was split by sex of parents which can explain why the results for the CFAs, bifactor, and Item Response Modeling were robust and replicated in this paper. Noteworthy, this study included fewer items that the original psychometric analyses [23, 24] to reduce farther burden. In this study, key considerations when finalizing the results was ease of interpretability, fit across both survey languages, and the need to remain grounded in the theoretical model [23, 24]. We observed that the hypothesized solutions were still validated and as such this paper provides brief tools for assessing key constructs of interests that are typically targeted by interventions. The less than optimal internal consistencies we observed for some parenting practices scales could make it more difficult to detect change unless the intervention has a large impact on these parenting practices. Cronbach’s alpha is influenced by sample size, so using the shortened version of the FPP and PAPP should be used with caution as a lower reliability can impact the ability to detect difference over time [56]. Thus, some cautions should be taken when using scales that have lower reliabilities unless the scale is augmented with other items.

Additional strengths of the current psychometric evaluation include adequate sample size of the English language surveys and the oversampled Spanish version, and the demonstration of a robust method to increase the use of data with imbalanced groups. We examined factor structures in surveys that were administered using two languages and ethnicities that are common in the US population, which makes the results more generalizable and inclusive.

### Limitations

Our findings are limited by the cross-sectional study design hence we cannot draw conclusions on the stability of the factor structures over time. The oversampling technique could introduce random error into the item responses, however the balanced bootstrap is a variance reduction technique that reduces simulation errors and increases the efficiency of the simulation [57]. As is for any factor analysis, there is a chance that parameter estimates could be biased due to model misspecification. While our study focused on examining the structural validity of the scales, we have not tested full invariance of the items. However, comparisons of the factors’ loadings suggest that full invariance may not be supported. While much of the factor structures were replicated in the English and Spanish survey administered to the father, it is important to note that wording of some items could explain the slight differences observed. Use of “not” in the question is perhaps important to eliminate when items are translated into another language. Further, additional assessment of the understandability of the items may be warranted in a new sample. It is important to assess the mode of survey delivery (online vs in-person) as a potential source of bias in addition to potential differences in survey responses introduced by survey language. Potential for social desirability may be higher when the questionnaire is completed in person, and this may have biased the results as well.

## Conclusions

This work builds on previously published psychometrics of a large item bank for improving measurement of PAPP and FPP, now with a focus on diverse fathers of different ethnicity and responding in two different languages. The focus of this study was to examine the psychometric properties of the scales; thus we have not tested full invariance of the items. However, comparisons of the factors’ loadings suggest that full invariance may not be supported, suggesting that scale scores may not have the same interpretation across groups. Minimal to no evidence of invariance was also observed by previous studies that have tested parenting practices for measurement invariance by ethnicity [45]. This study supports the use of a reduced version of the parenting practices when administering the survey in English and Spanish to Hispanic and non-Hispanic fathers in the US. Further research is needed to investigate the measurement of fathers’ parenting practices across ethnic groups. This should include studies to improve factor structures while balancing the improved internal consistencies of longer scales with the participant burden of such longer surveys.

## Supplementary Information

Additional file 1. Factor structure resulting from Confirmatory Factor Analysis of the food parenting practices among Hispanic and non-Hispanic fathers.

Additional file 2. Factor structure resulting from Confirmatory Factor Analysis of the physical activity parenting practices among Hispanic and non-Hispanic fathers.

Additional file 3. Factor structure resulting from Confirmatory Factor Analysis of the Structure domain of physical activity parenting practices among Hispanic and non-Hispanic fathers.

## Data Availability

The datasets generated and/or analyzed during the current study are not publicly available due to human subject privacy, but are available from the corresponding author on reasonable request if institutional data sharing agreements are established.

## Abbreviations

CFA: Confirmatory Factor Analysis
CFI: Comparative Fit Index
FPP: Food Parenting Practices
I-ECV: Item Expected Cross-Validation Index
PA: Physical Activity
PAPP: Physical Activity Parenting Practices
RMSEA: Root Mean Square Error of Approximation
SRMR: Standardized Root Mean Square Residual
US: United States
