# Supplementary material for: Factor structure of food and physical activity parenting practices among US fathers by ethnicity and survey language: a cross sectional study
Source: BMC Public Health. 2025 Oct 28;25:3625. doi: 10.1186/s12889-025-24584-1 (PMC12560397; doi:10.1186/s12889-025-24584-1)
Supplement: Supplementary file 1 — Additional file 1. Factor structure resulting from Confirmatory Factor Analysis of the food parenting practices among Hispanic and non-Hispanic fathers. [file 12889_2025_24584_MOESM1_ESM.pdf]

Additional File 1. Confirmatory Factor Analysis of the food parenting practices among Hispanic and non-Hispanic fathers

			**Hispanic (n=261)**	**Non-Hispanic (n=378)**
**Autonomy promotion domain**<br>In the PAST MONTH,		**Factors**<br>($\alpha_{Hispanic}$ / $\alpha_{Non\text{-}Hispanic}$)	**CFA**[1]<br>ʎ Factors	**CFA**[2]<br>ʎ Factors
1	on average how many times Did your child help you prepare dinner meals?	**Child Involvement** (.66 /.61)	0.55	0.45
2	on average how many times Did your child help you prepare vegetable dishes		0.73	0.56
3	on average how many times Did you give your child a choice of vegetables to eat at dinner?		0.59	0.34
7	how often did you Ask your child's opinion about what to make for meals?		0.43	0.43
29*	how often did you Give child a choice of how to season their fruit or vegetables		0.39	0.51
[1] (RMSEA=0.14 90%CI (0.09-0.19), CFI=0.94, SRMR=0.04); [2] (RMSEA=0.09 90%CI (0.06-0.15), CFI=0.97, SRMR=0.04). *Item 29 was added in this sample based on cognitive interviews with Latino fathers.				
**Structure domain**<br>In the PAST MONTH,		**Factors**<br>($\alpha_{Hispanic}$ / $\alpha_{Non\text{-}Hispanic}$)	**CFA**[3]<br>ʎ Factors	**CFA**[4]<br>ʎ Factors
9	how often did you keep sweet and salty treats out of your child's reach?	**Covert Control** (.52/.67)	0.30	0.34
10	how often did you hide soda and sugary drinks in places where your child could not find them?		0.63	0.59
14	how often did you throw away left over sweet or salty treats to discourage your child from eating them?		0.49	0.66
15	how often did you not bring soda or sweet drinks into your home?		0.49	0.72
16	how often did you eat or drink a healthy snack just because child was around?	**Modeling** (.69/.54)	0.72	0.59
21	how often did you eat healthy portions while in front of your child (for example take a smaller portion)		0.77	0.57
23	how often did you show how much you enjoyed eating vegetables while eating with your child?		0.62	0.53
	**Correlation between Covert Control & Modeling**		0.65	0.55

			Hispanic (n=261)	Non-Hispanic (n=378)
[3](RMSEA=0.10 90%CI (0.07-0.13), CFI=0.91, SRMR=0.06); [4](RMSEA=0.08 90%CI(0.06-0.11), CFI=0.94, SRMR=0.05)				
**Control domain** In the PAST MONTH,		**Factors** ($\alpha_{Hispanic}$ / $\alpha_{Non-Hispanic}$)	**CFA[5]** ʎ Factors	**CFA[6]** ʎ Factors
12	how often did you tell your child they will be punished if he or she eats a sweet or salty treat without asking you?	**Threats & bribes** (.77 /.82)	0.49	0.63
18	how often did you take away dessert as punishment for bad behavior?		0.63	0.69
20	how often did you threaten to send to room if your child refused to eat the vegetables you served?		0.79	0.88
24	how often did you tell your child he or she will get dessert only if he or she tasted the vegetables you served?		0.52	0.49
26**	how often did you take away TV or videogame time if your child did not finish his or her meal?		0.71	0.79
27	how often did you send your child to his or her room if they did not finish their meal?		0.78	0.88
28	how often did you reward your child with a sweet or salty treat for good behaviors?		0.48	0.48
[5]RMSEA=0.08 90%CI (0.05-0.11), CFI=0.96, SRMR=0.04; [6] (RMSEA=0.13 90%CI (0.11-0.16), CFI=0.93, SRMR=0.06). **Item 26 was adapted from item 11.				

α=Cronbach's alpha; ʎ Factor = Standardized factor loadings.
Item wording has been shortened to conserve space.
