# Supplementary material for: Factor structure of food and physical activity parenting practices among US fathers by ethnicity and survey language: a cross sectional study
Source: BMC Public Health. 2025 Oct 28;25:3625. doi: 10.1186/s12889-025-24584-1 (PMC12560397; doi:10.1186/s12889-025-24584-1)
Supplement: Supplementary file 2 — Additional file 2. Factor structure resulting from Confirmatory Factor Analysis of the physical activity parenting practices among Hispanic and non-Hispanic fathers. [file 12889_2025_24584_MOESM2_ESM.pdf]

Additional File 2. Confirmatory Factor Analysis of the physical activity parenting practices among Hispanic and non-Hispanic fathers

		**Factors** ($\alpha_{Hispanic}$ / $\alpha_{Non-Hispanic}$)	**Sub-Factors** ($\alpha_{Hispanic}$ / $\alpha_{Non-Hispanic}$)	**Hispanic (n=261)**		**Non-Hispanic (n=378)**	
**Autonomy promotion domain**<br><br>In the PAST MONTH,				**CFA[1]** ʎ Factors	**Bi-Factor[2]** I-ECV	**CFA[3]** ʎ Factors	**Bi-Factor[4]** I-ECV
3	how often did you make your child's sport or physical activity a conversation topic?	**Autonomy Support** (.86 / .83)	**Involvement** (.76/ .74)	0.80	0.77	0.74	0.81
5	how often did you teach your child sport or physical activity skills?			0.86	0.62	0.77	0.28
8	how often did you watch your child practice a sport or physical activity?			0.83	0.91	0.78	0.79
36	I find it stimulating to hear my child talk about the progress he/she is making in learning a new sport or physical activity skill.			0.43	0.95	0.49	1.00
10	how often did you praise your child for being physically active or for taking part in sports or physical activity classes?		**Praise** (.84/ .79)	0.85	0.90	0.84	0.92
11	how often did you tell your child he or she is doing well in his or her physical activity or sports?			0.93	0.96	0.88	0.93
	**Correlation between Involvement & Praise**			0.91		0.86	
[1](RMSEA=0.16 90%CI (0.12-0.19), CFI=0.95, SRMR=0.05); [2] (RMSEA=0.11 90%CI (0.05-0.17), CFI=0.99, SRMR=0.01)<br>[3] (RMSEA=0.15 90%CI (0.12-0.18), CFI=0.94, SRMR=0.05); [4](RMSEA=0.00 90%CI (0.00-0.06), CFI=1.00, SRMR=0.00)							
**Structure domain**<br><br>In the PAST MONTH,				**CFA[5]** ʎ Factors	**Bi-Factor[6]** I-ECV	**CFA[7]** ʎ Factors	**Bi-Factor[8]** I-ECV
1	how often did you play ball or sports with your child?	**Co-Participation / Modeling** (.90 / .86)	**Co-Participation** (.84/ .80)	0.78	0.53	0.83	0.42
2	how often did you ask your child to be active with you?			0.86	0.65	0.90	0.53
7	how often did you walk or bike with your child to go to places that are near your home even though it would be quicker to drive?			0.68	0.88	0.57	0.77

		Factors ($\alpha_{Hispanic}$ / $\alpha_{Non\text{-}Hispanic}$)	Sub-Factors ($\alpha_{Hispanic}$ / $\alpha_{Non\text{-}Hispanic}$)	**Hispanic (n=261)**		**Non-Hispanic (n=378)**	
12	how often did you arrange for your child to be with friends that would encourage your child to be physically active?			0.69	0.96	0.53	0.87
17	Select the best answer for you: Our family is physically active together.			0.78	0.99	0.75	0.74
9	how often did you do household chores in front of your child to show him/her you are physically active? (New)		**Modeling** (.83/ .75)	0.59	1.00	0.37	0.98
13	how often did you do at least 30 minutes of physical activity or exercise (e.g., walking, cycling, or playing a sport) on your own or with others?			0.73	1.00	0.70	0.95
20	Select the best answer for you: I am physically active in front of my child.			0.84	1.00	0.86	0.97
29	Select the best answer for you: I talk about my physical activity with my child.			0.80	0.99	0.70	0.66
33	Select the best answer for you: I tell my child how much I like to exercise or be physically active.			0.79	0.07*	0.68	0.71
	**Correlation between Co-participation & Modeling**			0.88		0.78	

[5](RMSEA=0.14 90%CI (0.12-0.15), CFI=0.89, SRMR=0.05); [6](RMSEA=0.09 90%CI (0.08-.0.12), CFI=.0.96, SRMR=0.04)
[7](RMSEA=0.13 90%CI (0.12-0.15), CFI=0.88, SRMR=0.07); [8](RMSEA=0.10 90%CI (0.08-0.12), CFI=0.95, SRMR=0.05)
*Item 33 I-ECV is low because the factor loading was >1 in absolute value (2.47) thus we tested a one factor solution with correlated error terms between items 1 and 2, items 33 and 29, and items 20 and 17 and the model produced adequate fit (see Additional File 3).

**Control domain**<br><br>Select the best answer for you:				**CFA[9]** ʎ Factors	**Bi-Factor[10]** I-ECV	**CFA[11]** ʎ Factors	**Bi-Factor[12]** I-ECV
16	I try to guilt my child to be more physically active by telling him/her that he or she has been lazy.	**Coercive Control** (.91/.89)	**Guilt** (.82/.79)	0.88	0.95	0.84	0.98
21	I tell my child he or she will gain weight if he or she doesn't exercise.			0.71	0.53	0.68	0.77

		Factors ($\alpha_{Hispanic}$ / $\alpha_{Non\text{-}Hispanic}$)	Sub-Factors ($\alpha_{Hispanic}$ / $\alpha_{Non\text{-}Hispanic}$)	**Hispanic (n=261)**		**Non-Hispanic (n=378)**	
32	I show my child people who are overweight as a way to get him or her to be more physically active.			0.71	0.55	0.74	0.70
34	To make my child do more physical activity, I tell him or her to stop being lazy.			0.87	0.76	0.87	0.81
15	I threaten to take away privileges (e.g., TV or video game times) if my child is not physically active in his or her free time.		**Pressure** (.87/.86)	0.65	0.79	0.77	0.84
18	I promise my child a sweet or salty treat (e.g., dessert) if he or she is active.			0.58	0.75	0.59	1.00
19	I complain to my child when he or she is not active enough.			0.74	0.81	0.78	0.99
24	The only way I can get my child to play outside is by insisting that my child goes outside.			0.78	0.99	0.73	0.71
25	I get upset and angry at my child if he or she is not physically active in his or her free time.			0.85	1.00	0.86	0.78
26	I have to push my child hard to get better at sports or physical activity skills.			0.70	0.87	0.66	0.60
27	When the weather allows, I force my child to play outside even if he or she does not feel like it.			0.77	0.77	0.70	0.49
35	The only way I get my child to be physically active in his or her free time is by forcing him/her to be active.			0.89	0.94	0.84	0.84
	**Correlation between Guilt & Pressure**			0.91		0.83	

[9](RMSEA=0.13 90%CI (0.12-0.15), CFI=0.89, SRMR=0.05); [10](RMSEA=0.17 90%CI (0.15-0.19), CFI=0.87, SRMR=0.06); [11](RMSEA=0.12 90%CI (0.10-0.13), CFI=0.91, SRMR=0.06); [12](RMSEA=0.14 90%CI (0.12-0.15), CFI=0.90, SRMR=0.07)

α=Cronbach’s alpha; ʎ Factor = Factor loadings; I-ECV = Explained Common Variance for a given item, where I-ECV > .50 indicated the proportion of common variance due to the general domain. Items with I-ECV < .50 were kept if it made sense conceptually and was supported in one of the samples.
