# Supplementary material for: Factor structure of food and physical activity parenting practices among US fathers by ethnicity and survey language: a cross sectional study
Source: BMC Public Health. 2025 Oct 28;25:3625. doi: 10.1186/s12889-025-24584-1 (PMC12560397; doi:10.1186/s12889-025-24584-1)
Supplement: Supplementary file 3 — Additional file 3. Factor structure resulting from Confirmatory Factor Analysis of the Structure domain of physical activity parenting practices among Hispanic and non-Hispanic fathers. [file 12889_2025_24584_MOESM3_ESM.pdf]

Additional File 3. Confirmatory Factor Analysis, Structure domain (physical activity parenting practices) among Hispanic and non-Hispanic fathers

		Factors ($\alpha_{Hispanic}$ / $\alpha_{Non\text{-}Hispanic}$)	Hispanic (n=261)	Non-Hispanic (n=378)
**Structure domain** In the PAST MONTH,			**CFA**[1] ʎ Factors	**CFA**[2] ʎ Factors
1	how often did you play ball or sports with your child?	**Co-Participation / Modeling** (.90 / .86)	0.67	0.67
2	how often did you ask your child to be active with you?		0.78	0.76
7	how often did you walk or bike with your child to go to places that are near your home even though it would be quicker to drive?		0.67	0.59
12	how often did you arrange for your child to be with friends that would encourage your child to be physically active?		0.72	0.56
17	Select the best answer for you: Our family is physically active together.		0.77	0.76
9	how often did you do household chores in front of your child to show him/her you are physically active? (New)		0.62	0.39
13	how often did you do at least 30 minutes of physical activity or exercise (e.g., walking, cycling, or playing a sport) on your own or with others?		0.76	0.68
20	Select the best answer for you: I am physically active in front of my child.		0.79	0.84
29	Select the best answer for you: I talk about my physical activity with my child.		0.75	0.62
33	Select the best answer for you: I tell my child how much I like to exercise or be physically active.		0.71	0.58

[1](RMSEA=0.07 90%CI (0.05-0.09), CFI=0.97, SRMR=0.03); [2](RMSEA=0.11 90%CI (0.09-.0.12), CFI=.0.93, SRMR=0.05). Correlated error terms were specified between items 1 and 2, items 33 and 29, and items 20 and 17. α=Cronbach's alpha; ʎ Factor = Factor loadings.
